# A Three-Dimensional Time-Varying Channel Model for THz UAV-Based Dual-Mobility Channels

**DOI:** 10.3390/e26110924

**Published:** 2024-10-30

**Authors:** Kai Zhang, Fenglei Zhang, Yongjun Li, Xiang Wang, Zhaohui Yang, Yuanhao Liu, Changming Zhang, Xin Li

**Affiliations:** 1 School of Information and Navigation, Air Force Engineering University, Xi’an 710077, China; 13262733921@163.com (K.Z.); 15029889024@163.com (F.Z.); liu_yuanhao@outlook.com (Y.L.); 18829443976@163.com (X.L.); 2 College of Information Science and Electronic Engineering, Zhejiang University, Hangzhou 310027, China; yang_zhaohui@zju.edu.cn; 3 Research Center of Novel Computational Sensing and Intelligent Processing, Zhejiang Lab, Hangzhou 311121, China; zhangcm@zhejianglab.com

**Keywords:** Time-varying channel, terahertz (THz), unmanned aerial vehicle (UAV), channel model, path loss, time auto-correlation function (T-ACF), Doppler power spectrum density (DPSD)

## Abstract

Unmanned aerial vehicle (UAV) as an aerial base station or relay device is a promising technology to rapidly provide wireless connectivity to ground device. Given UAV’s agility and mobility, ground user’s mobility, a key question is how to analyze and value the performance of UAV-based wireless channel in the terahertz (THz) band. In this paper, a three-dimensional (3D) time-varying channel model is proposed for UAV-based dual-mobility wireless channels based on geometric channel model theory in THz band. In this proposed channel model, the small-scale fading (e.g., scattering fading and reflection fading) on rough surfaces of communication environment and the atmospheric molecule absorption attenuations are considered in THz band. Moreover, the statistical properties of the proposed channel model, including path loss, time autocorrelation function (T-ACF) and Doppler power spectrum density (DPSD), have been derived and the impact of several important UAV-related and vehicle-related parameters have been investigated and compared to millimeter wave (mm-wave) band. Furthermore, the correctness of the proposed channel model has been verified via simulation, and some useful observations are provided for the system design of THz UAV-based dual-mobility wireless communication systems.

## 1. Introduction

The space-air-ground integrate networks (SAGINs) and THz communication technology as the key technologies of the sixth-generation (6G) mobility communication technology, they have gained significant attention in recent years. SAGIN can provide seamless connectivity for future communication systems, THz communication technology can provide ultra-high data rate and ultra-wideband. With the advantages of high flexibility, rapid 3-D deployment, low cost and high mobility, unmanned aerial vehicles (UAVs) can be deployed in the air to increase the probability of Line-of-Sight (LoS) transmissions when working as a relay to establish wireless communication links between the mobile devices (MDs) on the ground and BSs. In SAGINs, UAV is widely used in many scenarios [1] serving as air platforms (e.g., base station and relay station) for the wireless communication systems, and UAV is a promising technology to rapidly provide wireless connectivity to ground mobile/stationary devices or air device. Therefore, it is foreseen that the future UAV-based communication systems can support ultra-high data rate [2], which requires ultra-large transmission bandwidth. For the THz band, it has rich spectrum resources, which can meet the needs of ultra-high date rate. The channel model is essential for wireless communication technology in the THz bands. It accurately characterizes UAV-based wireless channels and is helpful to system design and performance evaluation of UAV-based THz wireless communications. However, so far, there are limited studies on channel modeling for UAV-based communications in the THz band.

The UAV has several important characteristics that make it the ideal choice for some application scenarios. These include rapid three-dimensional (3D) deployment, low cost, and high mobility. It can serve as both an air base station and a relay station, and can be used in various fields including commercial and military domains [3,4,5,6,7,8]. In general, UAVs have two main applications in wireless communications: air-to-ground (A2G) [9,10,11,12,13] and air-to-air (A2A) [11,14]. The existing literature clearly shows that the time-varying channel properties of the wireless channel of UAV-based communication, including the number of paths, average fading duration (AFD), space-time correlation (STC), path gain, Doppler power spectrum density (DPSD), and dynamic angle parameters, have been extensively investigated in different scenarios and carrier frequencies (e.g., microwave [9,10] and millimeter wave [11,12,13] bands).

Meanwhile, the data rate requirement for wireless communication via UAVs is increasing. The terahertz (THz) band, ranging from 0.1 THz to 10 THz, offers significant benefits due to its huge bandwidth. This facilitates ultra-high data transmission rates in various wireless applications. The peak data rates of 160 Gb/s and 1.176 Tbps have been achieved for THz wireless communications in [14,15]. The existing literature clearly shows that studies of THz and sub-THz wireless channels have been carried out both indoors and outdoors. These studies have involved measurements [16,17,18], channel modeling [16,17,19,20], and performance analysis [18,19,20]. Then, the statistical properties of the wireless channel have been investigated, including the path loss, scattering attenuation [18], and space-time-frequency correlation (STFC) [19]. The hybrid dynamic channel model and non-stationary space-time-frequency channel model in the THz band were proposed in [20]. Additionally, the authors from [20] take into consideration atmospheric absorption attenuations. The authors in [21] proposed a THz channel model for indoor scenarios based on ray tracing. They studied the characteristics of propagation paths with LoS paths, reflection paths, scattering paths, and diffraction paths.

In addition, several studies have been conducted on the channel modeling of UAV-based wireless communication in sub-THz bands [22,23,24]. The existing literature has investigated the three-dimensional (3D) time-varying channel model of UAV-based wireless communication for the A2G and A2A scenarios. However, there have been limited studies on the UAV-based wireless channels in the THz band. Furthermore, these studies only consider the impact of reflection and scattering fading on rough surfaces, but not the impact of atmospheric absorption attenuation.

In this paper, we present a novel channel model for THz UAV-based dual-mobility channels. This proposed model accurately characterizes the time-varying dual-mobility scenario in the THz UAV-based channels by combining the geometry-based stochastic model (GBSM), reflection and scattering fading, and atmospheric absorption attenuation. In addition, we have investigated the impact of some important time-varying UAV-related parameters on the channel characteristics, including the path loss, time autocorrelation function (T-ACF), and DPSD.

## 2. Modeling of Uav-Based Dual-Mobility Wireless Channels in the Thz Band

### 2.1. Description of the Wireless Communication System

**Figure 1:**
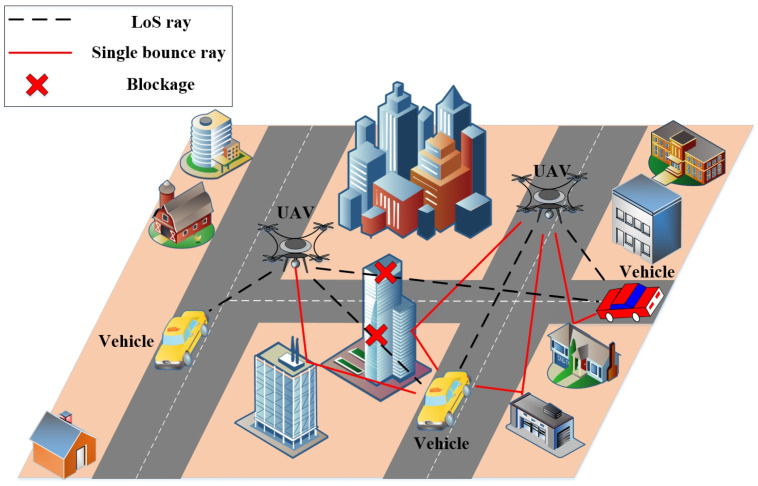
Real UAV-based dual-mobility wireless communications scenario in the THz band.

**Figure 2:**
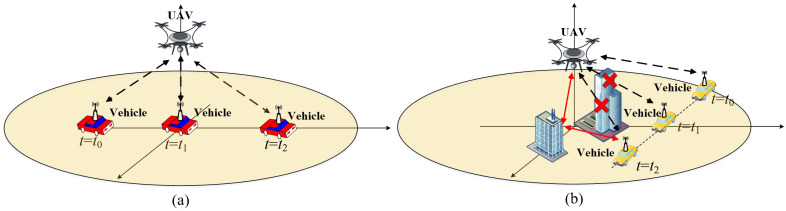
Different propagation paths between UAV and vehicle in time-varying UAV-based wireless communication system in the THz band: (**a**) LoS propagation path survival; (**b**) LoS propagation path death and NLoS propagation path birth.

This paper assumes that the antennas at the transmitter (Tx) and receiver (Rx) for the considered UAV-based wireless communication system are isotropic [18]. The antennas at Tx and Rx are located on the UAV and vehicle, respectively. The UAV-based dual-mobility communication system exhibit several characteristics, including 3D random deployment and mobility, flexible environment adaptability, and high space-time variation. The real UAV-based dual-mobility wireless communications scenario in the THz band is shown in Figure 1, where the propagation paths between UAV and vehicle are time-varying. In a time-varying wireless channel, the multipaths are informed in wireless environments with free space, obstructions, and rough surfaces. In general, the propagation path can be categorized into line-of-sight (LoS), and non-line-of-sight (NLoS) cases, as shown in Figure 2.

The height of Tx is always higher than that of Rx, so the scattering and reflection propagations around the vehicles are more than those around the UAV. Furthermore, the wavelength of the THz band is smaller than that of the mm-wave and microwave bands. This means that the probability of scattering and reflection on the rough surface of different materials in the THz band is greater than that in the mm-wave and microwave bands. Besides, the atmospheric molecule absorption attenuation of time-varying wireless channel $A_{\mathrm{ma}}\left(f_{c},t\right)$ increases rapidly with the increasing of the radio-wave frequency. In particular, $A_{\mathrm{ma}}\left(f_{c},t\right)$ in the THz band is higher than that in the mm-wave and micro-wave bands obviously. In this paper, $f_{c}$ denotes radio-wave frequency.

In signal processing and wireless communications [13], the output signal y(t) of a wireless communciation system is a function of the channel impulse response (CIR) h(t) and an input signal x(t), plus some random noise n(t), which can be expressed as y(t)=x(t)∗h(t)+n(t).

The CIR in the time-varying channels can be further expressed as a function of delay τ(t), and Doppler frequency shift $f_{D}\left(t\right)$, which can be written as:

(1) $$\begin{array}{c} \begin{array}{c} h\left(t\right)=\sum_{i=1}^{I(t)}\alpha_{i}\left(t\right)e^{j[2\pi tf_{D,i}\left(t\right)-2\pi f_{c}\tau_{i}\left(t\right)]} \end{array} \end{array}$$

where I(t) is the number of the time-varying multipath components (MPCs), $\alpha_{i}\left(t\right)$ is the channel gain value of the time-varying path, $\tau_{i}\left(t\right)$ is the delay of the time-varying path. In our modeling process, we consider the LoS, reflection, and scattering paths in the channel model to be separate.

### 2.2. Time-Varying Small-Scale Fading Coefficient of the NLoS Path on the Rough Surfaces

This paper precisely describes the time-varying small-scale fading coefficients of the NLoS path on rough surfaces for UAV-based dual-mobility wireless communication in the THz band. It considers both reflection and scattering paths on rough surfaces.

According to the Kirchhoff scattering theory [19] and the modified Beckmann-Kirchhoff theory [19,21], the reflection coefficient $R(f_{c},t)$ and scattering coefficient $S(f_{c},t)$ for a rough surface can be calculated as:

(2) $$\begin{array}{c} \begin{array}{cc} R(f_{c},t) & =-e^{\frac{-2\cos\theta_{i}\left(t\right)}{\sqrt{n_{t,\mathrm{ref}}^{2}-1}}}\;\cdot \;e^{-\frac{8\pi^{2}f_{c}^{2}\sigma_{h,\mathrm{ref}}^{2}\cos^{2}\left[\theta_{i}\left(t\right)\right]}{c^{2}}} \end{array} \end{array}$$

(3) $$\begin{array}{c} \begin{array}{cc} S(f_{c},t) & =-e^{\frac{-2\cos\theta_{1}\left(t\right)}{\sqrt{n_{t,\mathrm{sca}}^{2}-1}}}\;\cdot \;{\left[1+g\left(f_{c},t\right)+\frac{g^{2}\left(f_{c},t\right)}{2}+\frac{g^{3}\left(f_{c},t\right)}{6}\right]}^{-\frac{1}{2}}\\ \; & \times {\left\{\rho_{0}^{2}\left(f_{c},t\right)+\frac{\pi \cos\theta_{1}\left(t\right)}{100}\;\cdot \;\left[g\left(f_{c},t\right)e^{-v_{xy}\left(f_{c},t\right)}+\frac{g^{2}\left(f_{c},t\right)}{4}\;\cdot \;e^{-\frac{v_{xy}\left(f_{c},t\right)}{2}}\right]\right\}}^{\frac{1}{2}} \end{array} \end{array}$$

where $n_{t,\mathrm{ref}}$ and $n_{t,\mathrm{sca}}$ are the refractive indexes in reflection and scattering propagations respectively, $\sigma_{h,\mathrm{ref}}$ is the standard deviation in the height of rough surface in reflection propagation, which is commonly considered to be Gaussian distributed, *c* denotes the light speed, $\theta_{i}\left(t\right)$ and $\theta_{1}\left(t\right)$ are the angles of incident wave in reflection and scattering propagations respectively [19,21]. Detailed computation for the parameters including $g(f_{c},t)$, $\rho_{0}\left(f_{c},t\right)$ and $v_{xy}\left(f_{c},t\right)$ can be referred to [19].

### 2.3. Atmospheric Molecule Absorption Attenuation of Radio-Wave for the Wireless Channel

It is clear that water vapor and oxygen molecules have a much greater impact on the absorption attenuation of radio waves than other molecules [25]. This paper considers only the absorption attenuation caused by oxygen and water vapor molecules in the atmosphere. Then, according to the attenuation experience model of oxygen and water vapor molecules in ITU-R P.676-11 [25], the atmospheric molecule absorption attenuation can be described as:

#### 2.3.1. Attenuation Coefficient of Oxygen Molecule to Radio-Wave $\gamma_{o}\left(f_{c}\right)$

The frequency of radio-wave $f_{c}$ is from 100 GHz to 350 GHz, the atmospheric pressure *P* is 1013 hpa, and the temperature of environment *T* is 15 °C, then $\gamma_{o}\left(f_{c}\right)\left[\mathrm{dB}/\mathrm{km}\right]$ can be calculated by [25]:

(4) $$\begin{array}{c} \begin{array}{cc} \gamma_{o}\left(f_{c}\right) & =\left[3.79\times 10^{-7}f_{c}+\frac{0.265}{{\left(f_{c}-63\right)}^{2}+1.59}+\frac{0.028}{{\left(f_{c}-118\right)}^{2}+1.47}\right]\\ \; & \times {\left(f_{c}+198\right)}^{2}\times 10^{-3} \end{array} \end{array}$$

#### 2.3.2. Attenuation Coefficient of Water Vapor Molecule to Radio-Wave $\gamma_{w}\left(f_{c}\right)$

The frequency of radio-wave $f_{c}$ is from 100 GHz to 350 GHz, the atmospheric pressure *P* is 1013 hpa, and the temperature of environment *T* is 15 °C, then $\gamma_{w}\left(f_{c}\right)\left[\mathrm{dB}/\mathrm{km}\right]$ can be calculated by [25]:

(5) $$\begin{array}{c} \begin{array}{cc} \gamma_{w}\left(f_{c}\right) & =\left[0.067+\frac{3}{{\left(f_{c}-22.3\right)}^{2}+7.3}+\frac{9}{{\left(f_{c}-183.3\right)}^{2}+6}+\frac{4.3}{{\left(f_{c}-323.8\right)}^{2}+10}\right]\\ \; & \times f_{c}^{2}\rho_{w}\times 10^{-4} \end{array} \end{array}$$

When the environment temperature *T* is from −20 °C to +40 °C, the attenuation coefficient of oxygen and water vapor molecules can be expressed as [25]:

(6) $$\begin{array}{c} \begin{array}{cc} \; & \gamma_{o}^{\prime }\left(f_{c}\right)=\gamma_{o}\left(f_{c}\right)\left[1-0.01\times \left(T-15\right)\right] \end{array} \end{array}$$

(7) $$\begin{array}{c} \begin{array}{cc} \; & \gamma_{w}^{\prime }\left(f_{c}\right)=\gamma_{w}\left(f_{c}\right)\left[1-0.006\times \left(T-15\right)\right] \end{array} \end{array}$$

In UAV-based dual-mobility wireless communications, the heights of the transmitter and receiver are always different. To calculate atmospheric absorption attenuation, multiply the equivalent height of oxygen by the attenuation coefficient of the oxygen molecule and the equivalent height of water vapor by the attenuation coefficient of the water vapor. This is described in the ITU-R P.676-11 model theory [25]. Besides, the expression of atmospheric absorption attenuation varies with the elevations angle of the propagation path.

When the frequency range of radio-wave is from 100 GHz to 350 GHz, the equivalent heights of oxygen molecule and water vapor can be calculated as [25]:

(i) Oxygen molecule $O_{2}$:

(8) $$\begin{array}{c} \begin{array}{c} h_{o}\left(f_{c}\right)=5.542-1.76414\times 10^{-3}f_{c}+3.05354\times 10^{-6}f_{c}^{2}+\frac{6.815}{{\left(f_{c}-118.75\right)}^{2}+0.321} \end{array} \end{array}$$

(ii) Water vapor $H_{2}O$:

(9) $$\begin{array}{c} \begin{array}{c} h_{w}\left(f_{c}\right)=1.65\times \left[1+\frac{1.61}{{\left(f_{c}-22.23\right)}^{2}+2.91}+\frac{3.33}{{\left(f_{c}-183.3\right)}^{2}+4.58}+\frac{1.9}{{\left(f_{c}-3235.1\right)}^{2}+3.34}\right] \end{array} \end{array}$$

where the unit of $f_{c}$ is GHz and the unit of height is km in Equations (4)–(9).

When the elevation angle range of the propagation path is from 0° to 5°, the atmospheric absorption attenuation can be calculated as [25]:

(10) $$\begin{array}{c} \begin{array}{cc} A_{\mathrm{ma}}^{\mathrm{LoS}}\left(f_{c},t\right) & =\gamma_{o}^{\prime }\left(f_{c}\right)\sqrt{h_{o}\left(f_{c}\right)}\left\{\frac{\sqrt{r_{e}+h_{1}}\;\cdot \;F\left[x_{1}\left(t\right)\right]e^{-\frac{h_{1}}{h_{o}\left(f_{c}\right)}}}{\cos[\theta (t)]}-\frac{\sqrt{r_{e}+h_{2}}\;\cdot \;F\left[x_{2}\left(t\right)\right]e^{-\frac{h_{2}}{h_{o}\left(f_{c}\right)}}}{\cos[\theta^{\prime }\left(t\right)]}\right\}\\ \; & +\gamma_{w}^{\prime }\left(f_{c}\right)\sqrt{h_{w}\left(f_{c}\right)}\left\{\frac{\sqrt{r_{e}+h_{1}}\;\cdot \;F\left[x_{1}\left(t\right)\right]e^{-\frac{h_{1}}{h_{w}\left(f_{c}\right)}}}{\cos[\theta (t)]}-\frac{\sqrt{r_{e}+h_{2}}\;\cdot \;F\left[x_{2}\left(t\right)\right]e^{-\frac{h_{2}}{h_{w}\left(f_{c}\right)}}}{\cos[\theta^{\prime }\left(t\right)]}\right\} \end{array} \end{array}$$

where $r_{e}$ denotes the efficient radius of the earth in km, $h_{1}$ denotes the smaller height between Tx and Rx, $h_{2}$ denotes the higher height between Tx and Rx, and θ denotes the elevation from $h_{1}$ to $h_{2}$. The functions of F(·), θ(t), $\theta^{\prime }\left(t\right)$, $x_{i}\left(t\right)$ and $x_{i}^{\prime }\left(t\right)$ are referred to [25].

When the elevation angle range of the propagation path is from 5° to 90°, the atmospheric absorption attenuation can be calculated as [25]:

(11) $$\begin{array}{c} \begin{array}{c} A_{\mathrm{ma}}^{\mathrm{LoS}}\left(f_{c},t\right)=\frac{\gamma_{o}^{\prime }\left(f_{c}\right)h_{o}^{\prime }\left(f_{c}\right)+\gamma_{w}^{\prime }\left(f_{c}\right)h_{w}^{\prime }\left(f_{c}\right)}{\sin[\theta (t)]} \end{array} \end{array}$$

where $h_{o}^{\prime }\left(f_{c}\right)$ and $h_{w}^{\prime }\left(f_{c}\right)$ denote the modified equivalent heights of the absorption attenuation of oxygen molecule and water vapor respectively, which are calculated as [25]:

(12) $$\begin{array}{c} \begin{array}{cc} h_{o}^{\prime }\left(f_{c}\right) & =h_{o}\left(f_{c}\right)\left[e^{-\frac{h_{1}}{h_{o}\left(f_{c}\right)}}-e^{-\frac{h_{2}}{h_{o}\left(f_{c}\right)}}\right] \end{array} \end{array}$$

(13) $$\begin{array}{c} \begin{array}{cc} h_{w}^{\prime }\left(f_{c}\right) & =h_{w}\left(f_{c}\right)\left[e^{-\frac{h_{1}}{h_{w}\left(f_{c}\right)}}-e^{-\frac{h_{2}}{h_{w}\left(f_{c}\right)}}\right] \end{array} \end{array}$$

### 2.4. Propagation Scenarios for the Time-Varying Channel

This paper categorizes propagation paths into two main types: LoS and NLoS (i.e., reflection and scattering propagation paths) scenarios, as shown in Figure 2a,b.

#### 2.4.1. LoS Propagation Path

In a time-varying LoS propagation channel of this paper, there are no obstructions between the propagation paths of Tx and Rx, and as shown in Figure 1 and Figure 2a. Then, the path loss $PL_{\mathrm{LoS}}$ and delay $\tau_{\mathrm{LoS}}$ of the LoS propagation path are calculated as:

(14) $$\begin{array}{c} \begin{array}{cc} \; & PL_{\mathrm{LoS}}\left(t\right)=20\log_{10}\left[\frac{4\pi d_{\mathrm{LoS}}\left(t\right)}{\lambda_{c}}\right] \end{array} \end{array}$$

(15) $$\begin{array}{c} \begin{array}{cc} \; & \tau_{\mathrm{LoS}}\left(t\right)=\frac{d_{\mathrm{LoS}}\left(t\right)}{c} \end{array} \end{array}$$

where $d_{\mathrm{LoS}}\left(t\right)$ is the 3D distance between Tx and Rx for the LoS propagation path, $\lambda_{c}=f_{c}/c$ is the wavelength of radio-wave, and *c* is the speed of light.

#### 2.4.2. NLoS Propagation Path

In a time-varying channel of this paper, besides the direct LoS propagation path, the NLoS propagation paths usually undergo reflection and scattering on the rough surface and obstruction before arriving at the Rx antenna array as shown in Figure 1 and Figure 2b. In this paper, we focus on the reflection and scattering on the rough surface for the NLoS propagation paths. Besides, every NLoS propagation path only experience single reflection or scattering before arriving at the Rx antenna, i.e., single bounce propagation. The properties of time-varying in NLoS channels can be characterized using the Cartesian coordinates of the Tx and Rx antenna, points of scattering and reflection. In addition, the moving directions of the Tx (i.e., moving angles of elevation and azimuth $\beta_{\mathrm{Tx}}$ and $\alpha_{\mathrm{Tx}}$) and Rx (i.e., moving angle of azimuth $\alpha_{Rx}$), the locations of point of reflection and scattering are assumed to be arbitrary in this paper.

Accordingly, the angles of reflection and scattering will also be time-varying in this paper. Given the Cartesian coordinates of Tx, Rx, points of reflection and scattering, the time-varying path length and delay of each reflected and scattered ray can be calculated respectively, in both elevation and azimuth planes. Then, the time-varying propagation path lengths of reflection at the *n*-th (n=1,…,N(t), N(t) denotes the time-varying number of efficient reflection points) reflection point and scattering path at the *m*-th (m=1,…,M(t), M(t) denotes the time-varying number of efficient scattering points) scattering point are calculated as $d_{\mathrm{NLoS},n}^{\mathrm{ref}}\left(t\right)=|d_{\mathrm{Tx},n}^{\mathrm{ref}}\left(t\right)|+|d_{\mathrm{Rx},n}^{\mathrm{ref}}\left(t\right)|$ and $d_{\mathrm{NLoS},m}^{\mathrm{sca}}\left(t\right)=|d_{\mathrm{Tx},m}^{\mathrm{sca}}\left(t\right)|+|d_{\mathrm{Rx},m}^{\mathrm{sca}}\left(t\right)|$ respectively, where $d_{\mathrm{Tx},n}^{\mathrm{ref}}\left(t\right)$ and $d_{\mathrm{Rx},n}^{\mathrm{ref}}\left(t\right)$ are the time-varying path lengths seen by the Tx and Rx for the reflection propagation path at the *n*-th reflection point, respectively; $d_{\mathrm{Tx},m}^{\mathrm{sca}}\left(t\right)$ and $d_{\mathrm{Rx},m}^{\mathrm{sca}}\left(t\right)$ are the time-varying path lengths seen by the Tx and Rx for the scattering propagation path at the *m*-th scattering point, respectively. Hence, the delays of arrival of the *n*-th reflected and *m*-th scattered rays can be calculated as $\tau_{\mathrm{NLoS},n}^{\mathrm{ref}}\left(t\right)=d_{\mathrm{NLoS},n}^{\mathrm{ref}}\left(t\right)/c$ and $\tau_{\mathrm{NLoS},m}^{\mathrm{sca}}\left(t\right)=d_{\mathrm{NLoS},m}^{\mathrm{sca}}\left(t\right)/c$, and the path loss of *n*-th reflection propagation path and *m*-th scattering propagation path can also be calculated as:

(16) $$\begin{array}{c} \begin{array}{cc} \; & PL_{\mathrm{NLoS},n}^{\mathrm{ref}}\left(t\right)=20\log_{10}\left[\frac{16\pi^{2}d_{\mathrm{Tx},n}^{\mathrm{ref}}\left(t\right)\;\cdot \;d_{\mathrm{Rx},n}^{\mathrm{ref}}\left(t\right)}{\lambda_{c}^{2}\;\cdot \;R_{n}\left(f_{c},t\right)}\right] \end{array} \end{array}$$

(17) $$\begin{array}{c} \begin{array}{cc} \; & PL_{\mathrm{NLoS},m}^{\mathrm{sca}}\left(t\right)=20\log_{10}\left[\frac{16\pi^{2}d_{\mathrm{Tx},m}^{\mathrm{sca}}\left(t\right)\;\cdot \;d_{\mathrm{Rx},m}^{\mathrm{sca}}\left(t\right)}{\lambda_{c}^{2}\;\cdot \;S_{m}\left(f_{c},t\right)}\right] \end{array} \end{array}$$

where $R_{n}\left(f_{c},t\right)$ and $S_{m}\left(f_{c},t\right)$ are the time-varying reflection coefficient at the *n*-th reflection propagation path and the time-varying scattering coefficient at the *m*-th scattering propagation path, respectively.

#### 2.4.3. Time Auto-Correlation Function

According to the Equation (2) and description of propagation path above this paper, we can assume that the LoS and NLoS paths are independent of each other, and the reflection and scattering paths in NLoS path are independent of each other, then the time-varying CIR from Tx to Rx can be written as:

(18) $$\begin{array}{c} h\left(t\right)=h_{\mathrm{LoS}}\left(t\right)+h_{\mathrm{NLoS}}^{\mathrm{ref}}\left(t\right)+h_{\mathrm{NLoS}}^{\mathrm{sca}}\left(t\right) \end{array}$$

where

(19) $$\begin{array}{c} \begin{array}{cc} h_{\mathrm{LoS}}\left(t\right) & =\sqrt{\frac{\Omega (t)K(t)}{K(t)+1}}\;\cdot \;e^{j\{2\pi t\left[f_{D,\mathrm{LoS}}^{\mathrm{Tx}}\left(t\right)+f_{D,\mathrm{LoS}}^{\mathrm{Rx}}\left(t\right)\right]-2\pi f_{c}\tau_{\mathrm{LoS}}\left(t\right)\}} \end{array} \end{array}$$

(20) $$\begin{array}{c} \begin{array}{cc} h_{\mathrm{NLoS}}^{\mathrm{ref}}\left(t\right) & =\sqrt{\frac{\Omega \left(t\right)\eta_{\mathrm{ref}}\left(t\right)}{N(t)[K(t)+1]}}\sum_{n=1}^{N(t)}\alpha_{\mathrm{NLoS},n}^{\mathrm{ref}}\left(t\right)\;\cdot \;e^{j\theta_{n}}\\ \; & \times e^{j\{2\pi t\left[f_{D,\mathrm{NLoS},n}^{\mathrm{Tx},\mathrm{ref}}\left(t\right)+f_{D,\mathrm{NLoS},n}^{\mathrm{Rx},\mathrm{ref}}\left(t\right)\right]-2\pi f_{c}\tau_{\mathrm{NLoS},n}^{\mathrm{ref}}\left(t\right)\}} \end{array} \end{array}$$

(21) $$\begin{array}{c} \begin{array}{cc} h_{\mathrm{NLoS}}^{\mathrm{sca}}\left(t\right) & =\sqrt{\frac{\Omega \left(t\right)\eta_{\mathrm{sca}}\left(t\right)}{M(t)[K(t)+1]}}\sum_{m=1}^{M(t)}\alpha_{\mathrm{NLoS},m}^{\mathrm{sca}}\left(t\right)\;\cdot \;e^{j\theta_{m}}\\ \; & \times e^{j\{2\pi t\left[f_{D,\mathrm{NLoS},m}^{\mathrm{Tx},\mathrm{sca}}\left(t\right)+f_{D,\mathrm{NLoS},m}^{\mathrm{Rx},\mathrm{sca}}\left(t\right)\right]-2\pi f_{c}\tau_{\mathrm{NLoS},m}^{\mathrm{sca}}\left(t\right)\}} \end{array} \end{array}$$

with the time-varying Doppler frequency shifts terms $f_{D,\mathrm{LoS}}^{\mathrm{Tx}}\left(t\right)$, $f_{D,\mathrm{NLoS},n}^{\mathrm{Tx},\mathrm{ref}}\left(t\right)$, $f_{D,\mathrm{NLoS},n}^{\mathrm{Rx},\mathrm{ref}}\left(t\right)$, $f_{D,\mathrm{NLoS},m}^{\mathrm{Tx},\mathrm{sca}}\left(t\right)$, and $f_{D,\mathrm{NLoS},m}^{\mathrm{Rx},\mathrm{sca}}\left(t\right)$.

In Equations (19)–(21), K(t) denotes the Rician factor, and we assumed that K(t)=K in this paper. $\Omega \left(t\right)=E[|h\left(t\right){|}^{2}]$ (where E[·] is the statistical expectation operation) is the propagation path power from Tx to Rx. $\eta_{\mathrm{ref}}\left(t\right)$ and $\eta_{\mathrm{sca}}\left(t\right)$ are the power allocation factors of the refletion propagation path and scattering propagation path and satisfying $\eta_{\mathrm{ref}}\left(t\right)+\eta_{\mathrm{sca}}\left(t\right)=1$. The random phases (i.e., $\theta_{n}$ and $\theta_{m}$) introduced by reflection and scattering paths are assumed to be independently and uniform randomly distributed in the interval of [−π, π).

According to Equations (2), (3), (16) and (17), the time-varying channel coefficients of the reflection component and the scattering component can be calculated as:

(22) $$\begin{array}{c} \begin{array}{cc} \; & \alpha_{\mathrm{NLoS},n}^{\mathrm{ref}}\left(t\right)=\frac{\frac{\lambda_{c}^{2}\;\cdot \;R_{n}\left(f_{c},t\right)}{16\pi^{2}d_{\mathrm{Tx},n}^{\mathrm{ref}}\left(t\right)\;\cdot \;d_{\mathrm{Rx},n}^{\mathrm{ref}}\left(t\right)}}{\sqrt{\frac{1}{N(t)}\sum_{n}^{N(t)}{\left[\frac{\lambda_{c}^{2}\;\cdot \;R_{n}\left(f_{c},t\right)}{16\pi^{2}d_{\mathrm{Tx},n}^{\mathrm{ref}}\left(t\right)\;\cdot \;d_{\mathrm{Rx},n}^{\mathrm{ref}}\left(t\right)}\right]}^{2}}} \end{array} \end{array}$$

(23) $$\begin{array}{c} \begin{array}{cc} \; & \alpha_{\mathrm{NLoS},m}^{\mathrm{sca}}\left(t\right)=\frac{\frac{\lambda_{c}^{2}\;\cdot \;S_{m}\left(f_{c},t\right)}{16\pi^{2}d_{\mathrm{Tx},m}^{\mathrm{sca}}\left(t\right)\;\cdot \;d_{\mathrm{Rx},m}^{\mathrm{sca}}\left(t\right)}}{\sqrt{\frac{1}{M(t)}\sum_{m}^{M(t)}{\left[\frac{\lambda_{c}^{2}\;\cdot \;S_{m}\left(f_{c},t\right)}{16\pi^{2}d_{\mathrm{Tx},m}^{\mathrm{sca}}\left(t\right)\;\cdot \;d_{\mathrm{Rx},m}^{\mathrm{sca}}\left(t\right)}\right]}^{2}}} \end{array} \end{array}$$

For the stochastic time-varying channel model of this paper, the large-scale parameters in two adjacent time intervals are highly correlated, thus this paper use an time autocorrelation function (T-ACF) ρ(t,Δt) to express the relationship between the large-scale parameters in two adjacent time intervals, which can be written as:

(24) $$\begin{array}{c} \begin{array}{cc} \rho (t,\Delta t) & =\frac{E[h\left(t\right)h{\left(t+\Delta t\right)}^{*}]}{\sqrt{{E[|h\left(t\right)|}^{2}{]E[|h\left(t+\Delta t\right)|}^{2}]}}\\ \; & =\frac{E[h\left(t\right)h{\left(t+\Delta t\right)}^{*}]}{\sqrt{\Omega (t)\;\cdot \;\Omega (t+\Delta t)}} \end{array} \end{array}$$

where [·]* denotes the complex conjugate.

### 2.5. Doppler Power Spectrum Density

In this subsection, the DPSD is derived by the Fourier transform of T-ACF, which is given as:

(25) $$\begin{array}{c} S_{\mathrm{Doppler}}\left(t,f\right)=\int_{-\infty }^{\infty }\rho \left(t,\Delta t\right)e^{-j2\pi f\Delta t}d\Delta t \end{array}$$

## 3. Numerical Results and Analysis

In this section, the performance of the proposed channel model is analyzed in terms of the path loss, T-ACF, and DPSD with different channel model parameters, including mobile properties of Tx and Rx (e.g., moving speed and moving time), horizontal distance from Tx to Rx, and carrier frequencies (e.g., mm-wave and THz bands). By comparison the simulation and theoretical results, the correctness of proposed channel model can be validated, in which the simulation of the proposed UAV-based dual-mobility wireless channel model are carried out by ray-tracing method. The simulation and theoretical parameters are set as follows: $\alpha_{\mathrm{Tx}}=\pi ,\alpha_{\mathrm{Rx}}=\beta_{\mathrm{Tx}}=$0 (where $\alpha_{\mathrm{Tx}}$, $\alpha_{\mathrm{Rx}}$, and $\beta_{\mathrm{Tx}}$ are the azimuth and elevation of moving angle for Tx and Rx respectively), $\eta_{\mathrm{ref}}$ = 0.7, $\eta_{\mathrm{sca}}$ = 0.3, $n_{t,\mathrm{ref}}=n_{t,\mathrm{sca}}$ = 2.2 (i.e., the material of the rough surface is concrete), $\sigma_{h,\mathrm{ref}}$ = 0.05 mm, $\sigma_{h,\mathrm{sca}}$ = 0.15 mm (where $\sigma_{h,\mathrm{sca}}$ is the standard deviation in the height of rough surface in reflection propagation) [22,23,25]. For the analysis of the performance difference between mm-wave and THz, 60 GHz, 200 GHz, and 300 GHz are chosen as the carrier frequencies of mm-wave and THz bands respectively.

**Figure 3:**
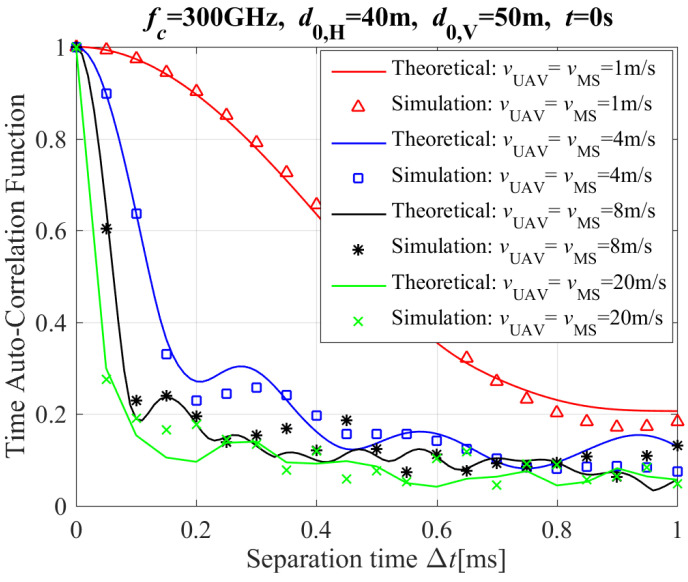
The T-ACF with different moving speeds of Tx and Rx for the NLoS path (including reflection and scattering paths).

Figure 3 shows the simulation and theoretical results of the T-ACF with different moving speeds of Tx and Rx for the NLoS propagation path, where $d_{0,V}$ and $d_{0,H}$ are the initial vertical and horizontal distances between UAV and MS (i.e., mobile station, MS denotes the moving vehicle on the ground), respectively. The results of Figure 3 clearly demonstrate that the faster speeds of the Tx and Rx, the quicker drop of T-ACF value, this conclusion is consistent with [11]. The reason is clear: the changing of CIR’s phase increases with the increase of the moving speed for the Tx and Rx. This means that the drop rate of T-ACF increases with the increase of Δt. Furthermore, the theoretical and simulation results for T-ACF are in close alignment. This is evident when comparing the theoretical results for T-ACF with the simulation results for T-ACF under the same configurations.

**Figure 4:**
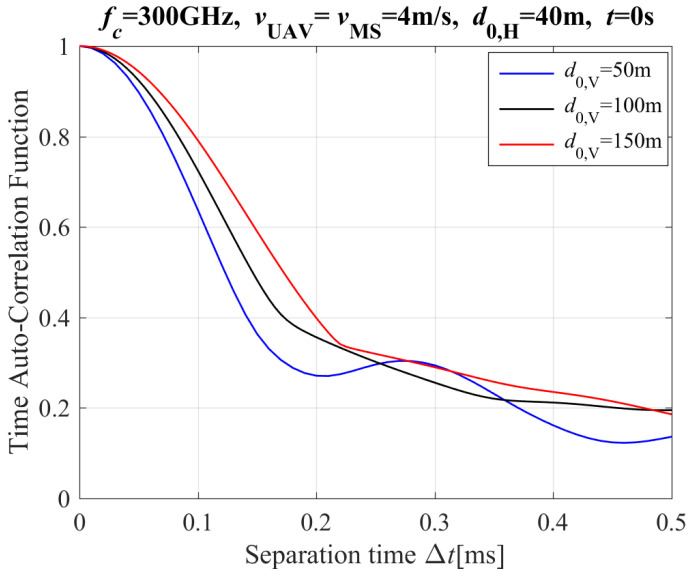
The T-ACF with different vertical distance of Tx and Rx for the NLoS path (including reflection and scattering paths).

Figure 4 shows the theoretical results of the T-ACF with different vertical distances between UAV and MS for the NLoS propagation path. The results of Figure 4 show that the T-ACF increases with an increase slowly of vertical distances between UAV and MS. The reason is clear: the slow changing of CIR’s phase increases with an increase slowly of vertical distances between UAV and MS.

**Figure 5:**
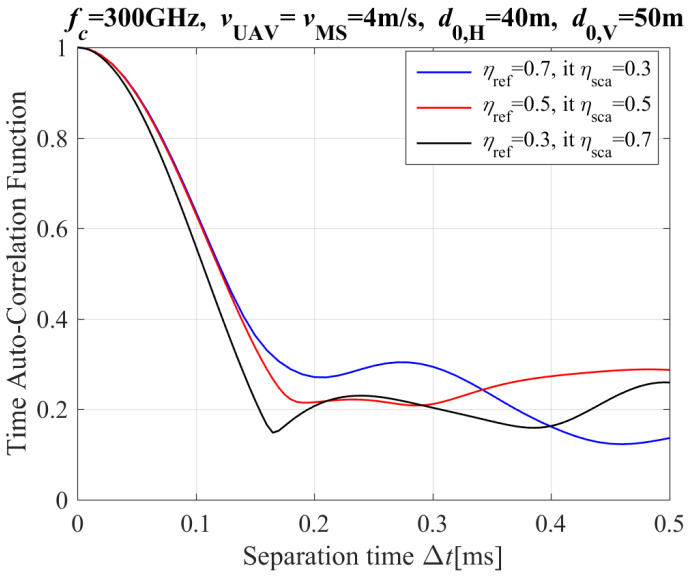
The T-ACF with different power ratio of reflection and scattering propagations for the NLoS path (including reflection and scattering paths).

Figure 5 depicts the theoretical results of the T-ACF with different power ratio between UAV and MS for the NLoS propagation path. According to Figure 4, it is found that the value of T-ACF with $\eta_{\mathrm{ref}}\geq$ 0.5 is more than that with $\eta_{\mathrm{ref}}<$ 0.5. Besides, the influence of $\eta_{\mathrm{ref}}$ and $\eta_{\mathrm{sca}}$ on T-ACF is not obvious.

**Figure 6:**
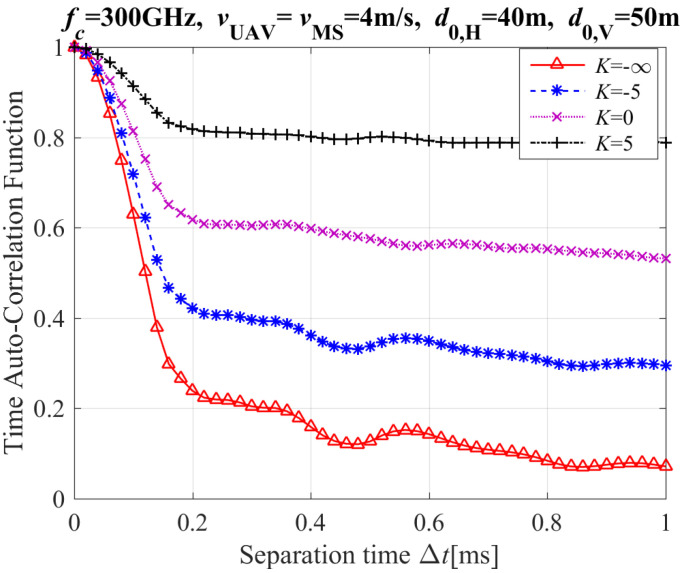
The T-ACF with different Ricican *K*-factor.

Figure 6 presents the T-ACF with different Ricican-*K* factors, where $d_{0,V}$ and $d_{0,H}$ are the initial vertical and horziontal distances between UAV and MS, respectively. Figure 6 clearly shows that the T-ACF increases with the Rician *K*-factor. These conclusions are in line with those in [11]. The Rician *K*-factor is defined as the ratio between the powers LoS path and NLoS path (i.e., reflection and scattering paths). Though not shown in this paper, it is found that the T-ACF’s value of LoS path is much higher than that of NLoS path. In addition, when the Rician *K*-factor decreases gradually, the power of NLoS path increases gradually and becomes more dominant. Thus, the value of T-ACF decreases.

**Figure 7:**
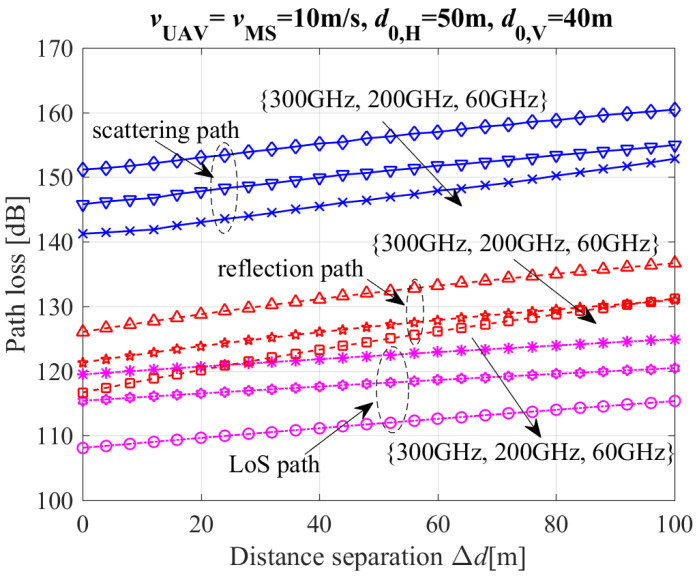
Path loss of the MPCs (including LoS, reflection, and scattering paths) with different carrier frequencies.

Figure 7 depicts the path loss of the MPCs (including LoS, reflection, and scattering paths) with different carrier frequencies, where $d_{0,V}$ and $d_{0,H}$ are the initial vertical and horizontal distances between UAV and MS, respectively. Figure 7 clearly shows that path loss increases with an increase in carrier frequency. The reason is that: (i) the atmospheric absorption attenuation is more noticeable in the higher frequency band than in the lower frequency band; (ii) the reflection and scattering fading in the higher frequency band is more serious than that in the lower frequency band. Therefore, the path loss in the higher frequency band is greater than that in the lower frequency band, as shown in Figure 7.

Besides, Figure 7 clearly shows that the path losses of NLoS paths (including scattering and reflection paths) are significantly higher than those of LoS paths in variant carrier frequencies. This is because that: (i) the propagation lengths for scattering and reflection paths are longer than those for the LoS path; (ii) the path loss of a line-of-sight (LoS) path is solely due to the spread attenuation in free space, the path loss of a non-line-of-sight (NLoS) path is the result of both the spread attenuation in free space and reflection and scattering fading on rough surfaces.

**Figure 8:**
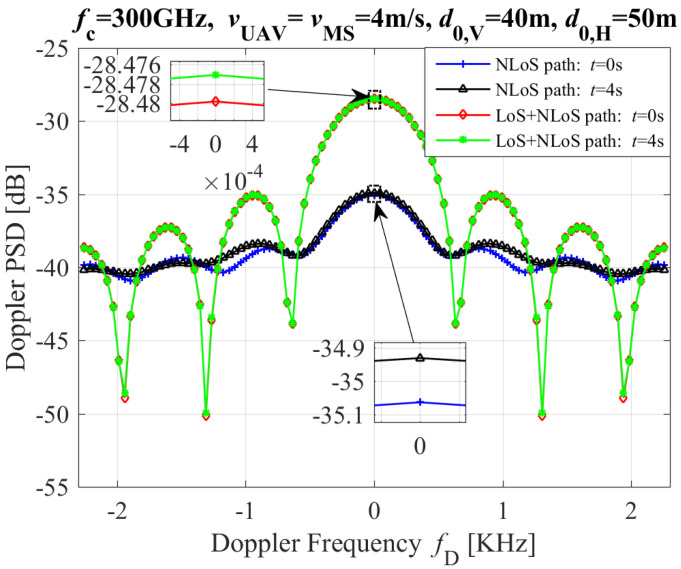
The DPSD with different moving times and different paths.

Figure 8 depicts the DPSD with different moving times and propagation paths (inluding NLoS and LoS+NLoS paths), where $d_{0,V}$ and $d_{0,H}$ are the initial vertical and horziontal distances between UAV and MS, respectively. From the Figure 8, we can see that the values of DPSDs for the LoS+NLoS path with variant moving times are greater than that the NLoS path in 300GHz band. In addition, the DPSDs’ values of both the LoS+NLoS and NLoS paths in t = 4 s are more than that in t = 0 s.

## 4. Conclusions

In this paper, we proposed a 3D time-varying channel model for UAV-based A2G dual-mobility wireless channels in the THz band, where the reflection and scattering fading on rough surfaces are considered, as well as atmospheric absorption attenuation of wireless propagation channels. In order to value assess the impact of UAV’s movement in 3D free space and the MS’s movement on the ground on performance of dual-mobility wireless channels, the path loss, T-ACF, and the DPSD are derived and subjected to comprehensive analysis. The correctness of the proposed model in this paper has been validated via simulation (i.e., ray tracing method). Numerical results have shown that the channel model parameters (e.g., moving speed, moving time, carrier frequency, and propagation path) at THz bands have significant impacts on path loss, T-ACF, and DPSD. The numerical results demonstrate that the path loss for different propagation paths (i.e., LoS, reflection, and scattering paths) exhibits an increase with the increase of carrier frequencies. Besides, the T-ACF decreases with an increase of the moving speed of UAV and MS. The influence of $\eta_{\mathrm{ref}}$ and $\eta_{\mathrm{sca}}$ on T-ACF is not obvious based on the results of T-ACF for the NLoS propagation path. In addition, the DPSD of NLoS+LoS path exhibits greater variability than that of LoS path for different Doppler frequencies.

## Data Availability

The original contributions presented in this study are included in the article. Further inquiries can be directed to the corresponding authors.

## Abbreviations

Abbreviations used in this Paper

**Abbreviation**	**Description**
UAV	Unmanned aerial vehicle
THz	Terahertz
3D	Three-dimensional
T-ACF	Time autocorrelation function
DPSD	Doppler power spectrum density
mm-wave	Millimeter wave
SAGNs	Space–air–ground integrate networks
6G	Sixth generation
A2G	Air to ground
A2A	Air to air
AFD	Average fading duration
STC	Space–time correlation
STFC	Space–time–frequency correlation
GBSM	Geometry-based stochastic model
Tx	Transmitter
Rx	Receiver
LoS	Line-of-sight
NLoS	Non-line-of-sight
CIR	Channel impulse response
MPCs	Multipath components
